# Consumer perceptions of safety in hospitals

**DOI:** 10.1186/1471-2458-6-41

**Published:** 2006-02-22

**Authors:** Sue M Evans, Jesia G Berry, Brian J Smith, Adrian J Esterman

**Affiliations:** 1 Department of Medicine, University of Adelaide, Adelaide, South Australia; 2 Centre for Injury Studies, Flinders University, Adelaide, South Australia; 3 Clinical Epidemiology and Health Outcomes Unit, The Queen Elizabeth Hospital, Adelaide, South Australia; 4 School of Nursing and Midwifery, University of South Australia, Adelaide, South Australia

## Abstract

**Background:**

Studies investigating adverse events have traditionally been principally undertaken from a medical perspective. The impact that experience of an adverse event has on consumer confidence in health care is largely unknown. The objectives of the study were to seek public opinion on 1) the rate and severity of adverse events experienced in hospitals; and 2) the perception of safety in hospitals, so that predictors of lack of safety could be identified.

**Methods:**

A multistage, clustered survey of persons residing in South Australia (2001), using household interviews (weighted n = 2,884).

**Results:**

A total of 67% of respondents aged over forty years reported having at least one member of their household hospitalised in the past five years; with the average being two hospital admissions in five years. Respondents stated that 7.0% (95%CI: 6.2% to 7.9%) of those hospital admissions were associated with an adverse event; 59.7% of respondents (95% CI: 51.4% to 67.5%) rated the adverse event as really serious and 48.5% (95% CI: 40.4% to 56.8%)  stated prolonged hospitalisation was required as a consequence of the adverse event. Perception of safety in hospitals was largely affected   by the experience of an adverse event; really serious events were the   most significant predictor of lack of safety in those aged 40 years and over (RR 2.38; p<0.001).

**Conclusion:**

The experience of adverse events negatively impacted on public confidence in hospitals. The consumer-reported adverse event rate in hospitals (7.0%) is similar to that identified using medical record review. Based on estimates from other studies, self-reported claims of adverse events in hospital by consumers appear credible, and should be considered when developing appropriate treatment regimes.

## Background

An adverse event is defined as an unintended injury or complication which results in death, disability or prolonged hospitalisation, and is caused by healthcare management [1]. Studies over the previous two decades have used various strategies to investigate the epidemiology of adverse events; most relate to hospitalised patients and are undertaken from a medical perspective [2].

Traditionally, the patient perspective on adverse events is obtained through complaints and litigation, which provide a somewhat biased picture, and likely underestimates the number of people dissatisfied with medical care [3]. For example, the elderly who are most at risk of adverse events [4] are also the least likely to complain [5]. Although patient surveys are increasingly being developed as valid tools to assess many facets of care provision [6], they are rarely used in investigating adverse events in hospitals.

Consumer surveys undertaken in the US and Australia have canvassed the opinions of adults regarding patient safety issues in health care and experiences of medical error [7-10]. However limitations in survey design have prevented comparison with rates determined from medical record review, the method most often cited to determine adverse event rates. We do not know whether the adverse event rate determined by consumers is congruent with that identified through medical record review undertaken by medical and nursing staff.

The aims of this study were to seek public opinion on:

1. the rate and severity of adverse events experienced in hospitals, using a lay definition, and

2. the perception of safety in hospitals

## Methods

### Data source

The data for this study were collected by household-based personal interview between September and November, 2001 [11]. The survey consisted of a multistage, systematic, clustered area sample (based on collector districts used by the Australian Bureau of Statistics in the 1996 Census [12]) of people aged 18 years or older living in metropolitan Adelaide, South Australia and country centres with a population exceeding 1000 (unweighted n = 2,945, weighted n = 2,884). The person in each household who had most recently had a birthday was interviewed. The survey was designed to have sufficient numbers to achieve a minimum of ± 1.75% accuracy with 95% confidence for any questionnaire item. Consent to participate in the survey was voluntary; no financial incentives were offered and interviewers obtained verbal consent prior to asking any questions of respondents.

### Survey design

To determine the rate and severity of adverse events experienced in hospitals, respondents were asked, 'In the last five years, how many times have you and members of your current household been admitted to hospital?'. If there had been a hospitalisation they were then asked, 'With regard to those hospitals stays, did anything ever go wrong that you thought might have been due to the way the health care was carried out?'. If the respondent answered in the affirmative, they were then directed to rate the severity of the adverse event(s) on a three-point Likert scale of (1) not serious, (2) a little serious and (3) really serious, and whether or not they thought it required extra time in hospital. Details of the total household size were collected from respondents to calculate a household hospital admission rate.

To ascertain public confidence in hospitals, respondents were asked, 'With regard to mistakes made in medical treatment, how safe would you feel being admitted to a public hospital?'. Responses were rated on a four-point Likert scale of (1) very safe, (2) pretty safe, (3) a little unsafe and (4) very unsafe. Respondent demographic details obtained included age, gender, metropolitan/country residence, annual household income, country of birth and Indigenous status (Aboriginal and/or Torres Strait Islander origin).

### Statistical analysis

A descriptive analysis was used to determine the adverse event rate and severity of the adverse event, with categorical variables reported as counts (percentages). To identify those most likely to have experienced an adverse event in their household and predictors of perceived lack of healthcare safety, univariate analyses were undertaken using weighted log binomial generalized linear modelling, followed by multivariate analysis aimed at determining the best joint predictors of safety. The conventional level of p ≤ 0.05 was taken to represent statistical significance.

Respondents who did not know how safe they felt being admitted to hospital were excluded from the analysis (representing 2.6% [n = 76] of all responses respectively). Analyses were weighted by age, sex and geographic region to be representative of the South Australian population (Table 1) [13]. The survey procedures of the Stata statistical software package were used [14].

## Results

From the initial 4,308 households selected randomly, 552 households were not contactable, 590 refused to be surveyed, 158 were either not available/too sick and 63 spoke no English. A total of 2,945 interviews were conducted (weighted n = 2,884), a participation rate of 78.4%. Table 1 shows the demographic profile of the respondents.

### 1. Consumer-reported adverse event rate and severity

**Figure 1:**
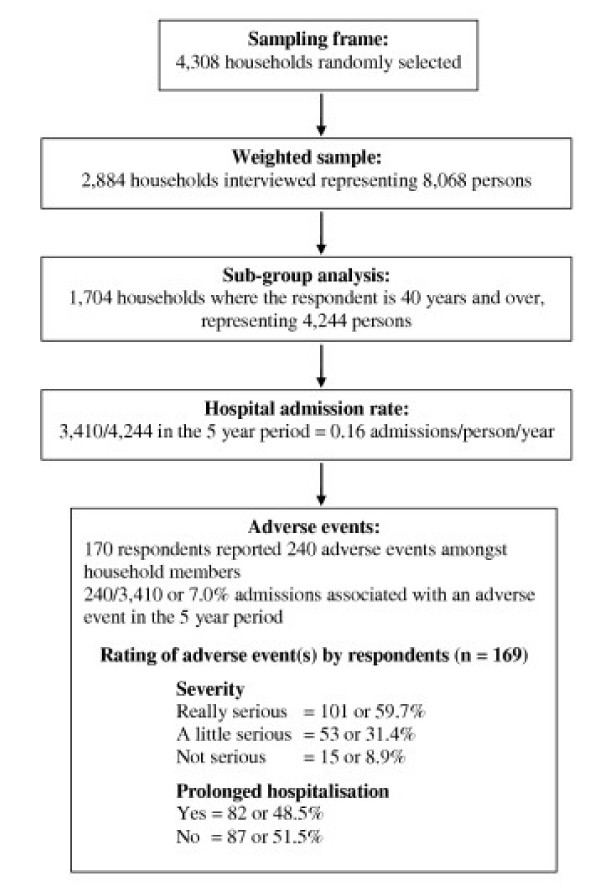
Flow diagram of adverse event rate and severity.

With regard to the consumer-reported adverse event rate, we chose to analyse only the findings for households in which the individual surveyed was aged 40 years and over. It was considered that respondents aged less than 40 years might be less likely to provide reliable information about the experience of an adverse event in their household, particularly when asked to recall events occurring five years prior to the survey. As questions asked were in relation to the entire household, the sample pool consisted of 4,244 people (Figure 1).

Of the 1,704 households surveyed where the respondent was aged 40 years or more, there were 1,137 (66.7%) where the respondent reported at least one hospital admission for any household member over the previous five years. This equated to 3,410 hospital admissions for all household members over the five year period. There were 11 respondents (6.5% of respondents aged 40 years and over) who did not know whether a household member had been admitted to hospital in the previous five years. Overall, 170 respondents (15.0%) reported that 240/3,410 or 7.0% (95%CI: 6.2% to 7.9%) of hospital admissions were associated with an adverse event over the five year period. When asked to rate the seriousness of the adverse event, 101 respondents (59.7%, 95% CI: 51.4% to 67.5%) rated the adverse event as really serious, and 82 (48.5%, 95% CI: 40.4% to 56.8%) indicated that extra time in hospital was required.

Tables 2 and 3 show the univariate and multivariate predictors of the likelihood of an adverse event occurring in the household of those aged 40 years and over. In Table 2, those more likely to have experienced one or more adverse events either to themselves or to a member of their household were aged less than 60 years or were an Indigenous Australian. In the multivariate model (Table 3), only being aged less than 60 years was a significant predictor.

### 2. Predictors of perceived lack of safety for respondents

To determine predictors of perceived lack of safety in hospitals, the total pool of 2,884 persons aged 18 years or older were interviewed. Of those interviewed, 5.2% (95% CI 4.4% – 6.1%) reported that they would feel very unsafe if admitted to hospital, 19.8% (95%CI 18.4% – 21.3%) stated that they would feel a little unsafe, 51.6% (95% CI 49.8% – 53.5%) stated that they would feel pretty safe and 23.3% (95% CI 21.8% – 24.9%) responded that they would feel very safe. Those who felt unsafe attending hospital were more likely to: be aged between 40 to 59 years, be female, reside in the metropolitan area, and have an annual household income greater than AUD $80,000 (Table 4). A strong predictor of feeling unsafe in hospital was having personally experienced an adverse event or knowing that a household member experienced an adverse event in the previous five years (Table 4). There was a gradient with the severity of the adverse event, i.e., the more severe the adverse event, the more the perception of lack of safety. In order to determine whether the experience of an adverse event affected respondents' perceptions of safety, multivariate analysis of those aged 40 years and over was undertaken. Multivariate analysis indicated that the best joint predictors for perceptions of lack of safety in public hospitals were being female, residing in a metropolitan area, having an income of between $20,000 and $80,000 and having either personally experienced a serious adverse event or being familiar with a household member who has (Table 5).

## Discussion

When people were asked to comment on how safe they felt going to hospital, one in four respondents felt either a little or very unsafe. Respondents aged forty years and over were asked how many times they or household members had been hospitalised in the previous five years. In each household there were on average two hospital admissions over the previous five years. Seven percent of those hospitalisations were associated with an adverse event. People most likely to report an adverse event in the household were aged less than sixty years. Over half of respondents regarded the adverse event as being really serious, and a third indicated that the adverse event had delayed discharge from hospital.

Individual experience of an adverse event or knowledge of a household member who had suffered one while hospitalised had a deleterious impact on consumer confidence in hospitals. The severity of the adverse event was the key factor in determining the extent to which respondents felt unsafe, with serious adverse events leading to approximately a two-fold increase in the likelihood that a person would feel unsafe in public hospitals.

The finding that a significant proportion of respondents felt unsafe attending hospitals may be attributable to a number of factors; people generally present to hospitals in the acute stage of their illness, are usually unfamiliar with the surroundings and personnel, and often receive complex and numerous procedures in a short period of time. People feeling less safe when aware of an adverse event having occurred in their household during a hospital admission is likely due to them having gained a greater understanding of the inherent risks associated with being hospitalised. The finding that people aged less than sixty years were more likely to report an adverse event compared to older respondents is contrary to findings in the literature [4]. It may be that older respondents were less aware of an adverse event occurring than younger people, or more accepting of adverse events as expected complications of their increasingly complex health problems or less likely to complain because they fear recrimination [5]. Results may be biased by the fact that young people are more likely than elderly people to have a larger household size.

Perceived or real past experience of an adverse event occurring to a household member may impact upon the patient-healthcare worker relationship, particularly in terms of patient confidence, and this may, in turn, negatively impact on attendance at follow-up appointments and treatment compliance. Recognising those people most likely to feel unsafe, particularly those who have experienced an adverse event, should assist healthcare providers in understanding human behaviour, thereby enabling management strategies to be developed and individually customised to address these concerns. This might include strategies such as providing hospital in the home services and focusing on early discharge with community support, where appropriate.

The adverse event rate of seven percent identified in this study is within the range of that identified by medical record review, which has estimated that between 2.9% [15] and 16.6% [1] of hospital admissions were associated with adverse events. Even the higher rate likely underestimates the true incidence, given that many adverse events are not recorded in medical records [16], and prospective studies have identified high incidences [17,18]. Although our survey was applied specifically to hospital-acquired adverse events, other consumer studies have found that adverse events in any setting leads to diminished perceived safety in the healthcare environment [7,9]

There were several limitations to the study. Firstly, the survey represents self-reported experiences by the public, using lay judgement of what constitutes an adverse event based on their interpretation of the definition provided. Respondents may not have construed this definition in the same way as medical reviewers, who used strict criteria. Secondly, there are inherent risks when using data based on a person's recall, namely limitation of the amount and type of information retained by people over time (recall bias). While respondents might have experienced more than one adverse event for the household, they were only asked to rate one of them. It may be the case that, for those who reported multiple adverse events, only the most severe adverse event was cited, resulting in an overestimate of severity and an underestimate of the adverse event rate. Thirdly, adverse event rates might be underestimated through respondents being unfamiliar with household members' medical history or because errors may have been concealed from them [19,20].

## Conclusion

The findings of this survey are of interest to public health professionals. Given that our consumer estimates of adverse events rates are similar to medical record review, claims of past adverse events by consumers would appear to be credible. If those who feel unsafe attending hospitals are themselves required to be hospitalised, strategies such as pre-admission hospital orientation and early discharge with hospital-in-the home services may assist in allaying fear. These and other strategies need to be considered when developing treatment regimes which best meet consumers' needs.

## Competing interests

The author(s) declare that they have no competing interests.

## Authors' contributions

Sue Evans has made substantial contribution to conception and design and interpretation of the data. She has written several drafts of the paper.

Jesia Berry has provided statistical analysis, has assisted in the conception and design and interpretation of the data, and has assisted with writing the manuscript.

Brian Smith has contributed to the conception and design, writing and interpretation of the data and has authorised funding for the study.

Adrian Esterman has provided statistical consultancy and has provided assistance in writing the manuscript including revising the draft in line with reviewers comments.

All authors read and approved the final manuscript.

## Pre-publication history

The pre-publication history for this paper can be accessed here:



## Figures and Tables

**Table 1:** Demographic profile of the respondent sample (weighted n = 2884), which was weighted by age, sex and geographical region to be comparable with the total population of South Australia in 2000 (n = 1,497,600) [13].

**Variable**	**Males**		**Females**		**Total**	
	**N**	**%**	**N**	**%**	**N**	**%**
**Age (yrs)**						
18–39	585	41.8	586	39.6	1171	40.6
40–59	499	35.6	505	34.1	1004	34.8
60+	318	22.7	391	26.4	709	24.6
Total	*1402*	*100.0*	*1482*	*100.0*	*2884*	*100.0*
**Residence**						
Country	428	30.5	433	29.2	861	29.9
Metropolitan	974	69.5	1049	70.8	2023	70.1
Total	*1402*	*100.0*	*1482*	*100.0*	*2884*	*100.0*
**Annual Household Income (AUD)**						
≤20,000	288	22.6	393	30.5	681	26.6
20,001–80,000	738	57.9	727	56.5	1465	57.2
80,001+	248	19.5	168	13.0	416	16.2
Total	*1274*	*100.0*	*1288*	*100.0*	*2562*	*100.0*
**Country of birth**						
Australia						
▪ Non-Indigenous	1011	72.2	1091	73.6	2102	72.9
▪ Indigenous	20	1.4	20	1.3	40	1.4
Europe						
▪ UK/Ireland	171	12.2	183	12.4	354	12.3
▪ Other	97	6.9	92	6.2	189	6.5
Asia	30	2.1	28	1.9	58	2.0
Other	72	5.1	68	4.6	140	4.9
Total	*1401*	*100.0*	*1482*	*100.0*	*2883*	*100.0*

**Table 2:** Respondents aged 40 years and over who perceived that an adverse event had occurred to themselves or a household member – univariate analysis.

**Variable**	**Adverse event (%)**	**Total (N)**	**RR**	**95% CI**	Sig.*
**Age (yr)**		*1126*			
40–59	17.3	648	1.00		
60+	12.2	478	0.70	0.52 – 0.96	0.025
**Gender**		*1126*			
Male	13.7	542	1.00		
Female	16.4	584	1.20	0.88 – 1.63	0.244
**Residence**		*1126*			
Rural	13.5	355	1.00		
Metropolitan	15.8	771	1.17	0.80 – 1.69	0.417
**Country of birth**		*1126*			
Australian: Non-Indigenous	14.0	763	1.00		
Australian: Indigenous	37.1	16	2.64	1.17 – 5.95	0.019
Europe: UK/Ireland	18.2	172	1.30	0.88 – 1.92	0.183
Europe: Other	15.8	113	1.12	0.67 – 1.88	0.654
Asia	8.4	15	0.60	0.09 – 3.91	0.595
Other	15.2	47	1.08	0.54 – 2.17	0.822
**Annual household income (AUD)**		*1018*			
<$20,00	15.6	382	1.00		
$20,001–$80,000	15.8	489	1.02	0.73 – 1.41	0.928
>$80,0001	15.6	147	1.00	0.59 – 1.69	0.996

* Weighted log binomial generalized linear model

**Table 3:** Respondents aged 40 years and over who perceived that an adverse event had occurred to themselves or a household member – multivariate analysis.

**Variable**	**RR**	**95% CI**	Sig.*
**Age (yr)**			
40–59	1.00		
60+	0.57	0.40 – 0.84	0.004
**Gender**			
Male	1.00		
Female	1.20	0.87 – 1.64	0.267
**Residence**			
Rural	1.00		
Metropolitan	1.16	0.79 – 1.71	0.454
**Country of birth**			
Australian: Non-Indigenous	1.00		
Australian: Indigenous	2.08	1.02 – 5.53	0.093
Europe: UK/Ireland	1.19	0.77 – 1.76	0.407
Europe: Other	1.06	0.60 – 1.78	0.844
Asia	0.58	0.09 – 3.64	0.553
Other	1.12	0.53 – 2.21	0.752
**Annual household income (AUD)**			
<$20,00	1.00		
$20,001–$80,000	0.83	0.57 – 1.20	0.322
>$80,0001	0.73	0.42 – 1.29	0.280

* Weighted log binomial generalized linear model

**Table 4:** Public perceptions of lack of safety in public hospitals – univariate analysis.

**Variable**	**Feel unsafe (%)**	**Total (N)**	**RR**	**95% CI**	Sig.*
**Age (yr)**		*2819*			
18–39	22.6	1157	1.00		
40–59	29.2	988	1.29	1.09 – 1.52	0.002
60+	23.2	674	1.02	0.85 – 1.23	0.797
**Gender**		*2819*			
Male	22.7	1381	1.00		
Female	27.3	1438	1.20	1.04 – 1.38	0.013
**Residence**		*2820*			
Country	18.2	844	1.00		
Metropolitan	28.0	1976	1.54	1.2 6 – 1.87	<0.001
**Country of birth**		*2818*			
Australia: Non-Indigenous	24.6	2067	1.00		
Australia: Indigenous	25.6	39	1.00	0.52 – 1.94	0.991
Europe: UK/Ireland	24.5	343	0.99	0.81 – 1.22	0.954
Europe: Other	28.5	179	1.15	0.88 – 1.50	0.294
Asia	24.6	57	1.03	0.53 – 1.99	0.935
Other	28.6	133	1.16	0.86 – 1.57	0.331
**Annual household income**		*2509*			
<$20,00	23.6	652	1.00		
$20,001–$80,000	24.5	1448	1.04	0.88 – 1.22	0.665
>$80,0001	30.8	409	1.30	1.04 – 1.62	0.019
**Previous hospital-acquired adverse event †**		*1091*			
No	21.6	923	1.00		
Yes	46.9	168	2.25	1.89 – 2.67	<0.001
**Severity of that hospital-acquired adverse event †**		*1091*			
No adverse event	21.6	923	1.00		
Not serious	29.2	15	1.06	0.49 – 2.23	0.898
A little serious	36.6	52	1.72	.1.36 – 2.36	0.001
Really serious	54.6	101	2.72	2.27 – 3.25	<0.001

* Weighted log binomial generalized linear model †respondents aged 40 years and over

**Table 5:** Public perceptions of lack of safety in public hospitals (respondents aged 40 years and over) – multivariate analysis.

**Variable**	**RR**	**95% CI**	Sig.*
**Age (yr)**			
40–59	1.00		
60+	0.80	0.61 – 1.03	0.085
**Gender**			
Male	1.00		
Female	1.28	1.02 – 1.60	0.032
**Residence**			
Rural	1.00		
Metropolitan	1.44	1.06 – 1.95	0.020
**Country of birth**			
Australian: Non-Indigenous	1.00		
Australian: Indigenous	0.50	0.18 – 1.40	0.187
Europe: UK/Ireland	0.83	0.60 – 1.14	0.251
Europe: Other	0.83	0.54 – 1.26	0.374
Asia	0.21	0.03 – 1.47	0.117
Other	1.07	0.63 – 1.80	0.806
**Annual household income (AUD)**			
<$20,00	1.00		
$20,001–$80,000	1.36	1.04 – 1.78	0.024
>$80,0001	1.20	0.80 – 1.81	0.385
**Severity of that hospital-acquired adverse event**			
No adverse event	1.00		
Not serious	1.36	0.67 – 2.76	0.396
A little serious	1.68	1.12 – 2.51	0.011
Really serious	2.38	1.85 – 3.07	<0.001

* Weighted log binomial generalized linear model
